# Delayed antibiotic prescribing for respiratory tract infections: individual patient data meta-analysis

**DOI:** 10.1136/bmj.n808

**Published:** 2021-04-28

**Authors:** Beth Stuart, Hilda Hounkpatin, Taeko Becque, Guiqing Yao, Shihua Zhu, Pablo Alonso-Coello, Attila Altiner, Bruce Arroll, Dankmar Böhning, Jennifer Bostock, Heiner C Bucher, Jennifer Chao, Mariam de la Poza, Nick Francis, David Gillespie, Alastair D Hay, Timothy Kenealy, Christin Löffler, David P McCormick, Gemma Mas-Dalmau, Laura Muñoz, Kirsty Samuel, Michael Moore, Paul Little

**Affiliations:** 1 Academic Unit of Primary Care, Population Sciences and Medical Education, Faculty of Medicine, University of Southampton, Southampton, UK; 2 Biostatistics Research Group, Department of Health Sciences, College of Life Sciences, University of Leicester, Leicester, UK; 3 Iberoamerican Cochrane Centre, Instituto de Investigación Biomédica Sant Pau (IIB Sant Pau-CIBERESP), Barcelona, Spain; 4 Institute of General Practice, Rostock University Medical Center, Rostock, Germany; 5 Department of General Practice and Primary Health Care, University of Auckland, Auckland, New Zealand; 6 Southampton Statistical Sciences Research Institute, University of Southampton, Southampton, UK; 7 Division of Health and Social Care Research, King’s College London, London, UK; 8 Basel Institute for Clinical Epidemiology and Biostatistics (CEB), University Hospital Basel and University of Basel, Switzerland; 9 Pediatric Emergency Medicine, State University of New York Downstate, Brooklyn, New York, USA; 10 Institut Català de la Salut, CAP Doctor Carles Ribas, Foc 112, Barcelona, Spain; 11 Centre for Trials Research, School of Medicine, College of Biomedical & Life Sciences, Cardiff University, Cardiff, UK; 12 Centre for Academic Primary Care, Population Health Sciences, Bristol Medical School, University of Bristol, Bristol, UK; 13 Department of Pediatrics, University of Texas Medical Branch at Galveston, Galveston, TX, USA; 14 Instituto de Investigación Biomédica Sant Pau (IIB Sant Pau), Barcelona, Spain; 15 Agència de Qualitat i Avaluació Sanitàries de Catalunya (AQuAS), Barcelona, Spain; 16 ASPIRE PPI Panel, Leeds Institute for Health Sciences, University of Leeds, Leeds, UK

## Abstract

**Objective:**

To assess the overall effect of delayed antibiotic prescribing on average symptom severity for patients with respiratory tract infections in the community, and to identify any factors modifying this effect.

**Design:**

Systematic review and individual patient data meta-analysis.

**Data sources:**

Cochrane Central Register of Controlled Trials, Ovid Medline, Ovid Embase, EBSCO CINAHL Plus, and Web of Science.

**Eligibility criteria for study selection:**

Randomised controlled trials and observational cohort studies in a community setting that allowed comparison between delayed versus no antibiotic prescribing, and delayed versus immediate antibiotic prescribing.

**Main outcome measures:**

The primary outcome was the average symptom severity two to four days after the initial consultation measured on a seven item scale (ranging from normal to as bad as could be). Secondary outcomes were duration of illness after the initial consultation, complications resulting in admission to hospital or death, reconsultation with the same or worsening illness, and patient satisfaction rated on a Likert scale.

**Results:**

Data were obtained from nine randomised controlled trials and four observational studies, totalling 55 682 patients. No difference was found in follow-up symptom severity (seven point scale) for delayed versus immediate antibiotics (adjusted mean difference −0.003, 95% confidence interval −0.12 to 0.11) or delayed versus no antibiotics (0.02, −0.11 to 0.15). Symptom duration was slightly longer in those given delayed versus immediate antibiotics (11.4 *v* 10.9 days), but was similar for delayed versus no antibiotics. Complications resulting in hospital admission or death were lower with delayed versus no antibiotics (odds ratio 0.62, 95% confidence interval 0.30 to 1.27) and delayed versus immediate antibiotics (0.78, 0.53 to 1.13). A significant reduction in reconsultation rates (odds ratio 0.72, 95% confidence interval 0.60 to 0.87) and an increase in patient satisfaction (adjusted mean difference 0.09, 0.06 to 0.11) were observed in delayed versus no antibiotics. The effect of delayed versus immediate antibiotics and delayed versus no antibiotics was not modified by previous duration of illness, fever, comorbidity, or severity of symptoms. Children younger than 5 years had a slightly higher follow-up symptom severity with delayed antibiotics than with immediate antibiotics (adjusted mean difference 0.10, 95% confidence interval 0.03 to 0.18), but no increased severity was found in the older age group.

**Conclusions:**

Delayed antibiotic prescribing is a safe and effective strategy for most patients, including those in higher risk subgroups. Delayed prescribing was associated with similar symptom duration as no antibiotic prescribing and is unlikely to lead to poorer symptom control than immediate antibiotic prescribing. Delayed prescribing could reduce reconsultation rates and is unlikely to be associated with an increase in symptoms or illness duration, except in young children.

**Study registration:**

PROSPERO CRD42018079400.

## Introduction

Antimicrobial resistance is an important public health concern.^1^ ^2^ The burden of antimicrobial resistance has increased substantially in recent years,^3^ and resistance to second and third line antibiotics is predicted to increase by 70% by 2030 if effective public health measures are not implemented.^4^

Reducing unnecessary and inappropriate use of antibiotics is crucial to reduce antimicrobial resistance, particularly in primary care where antibiotics are most prescribed.^2^ ^5^ However, antibiotics are commonly used to treat acute respiratory tract infections, despite studies showing that antibiotics have, at best, modest effects.^6^ ^7^ ^8^ ^9^ Guidelines recommend that the fewest number of antibiotic courses should be prescribed for the shortest period possible.^10^ ^11^ However, in the United Kingdom and internationally, antibiotics are still being overprescribed.^12^ ^13^ ^14^ ^15^ ^16^ Delayed antibiotic prescribing is a useful strategy that can be used to help reduce antibiotic use, especially during consultations when patients expect to receive an antibiotic prescription.^6^ A Cochrane review of 10 trials found that delayed prescribing was as effective as immediate prescribing in terms of clinical outcomes for cough and cold, but less effective for reducing fever, pain, and malaise in some studies, and with lower antibiotic use.^9^ However, the review noted a high level of heterogeneity between studies that made combining them in a traditional meta-analysis difficult, and did not allow sufficient power for the examination of subgroups of participants or complications.

These problems can be addressed in part by evidence synthesis using raw individual level data from relevant studies.^17^ ^18^ Therefore, we conducted a collaborative individual patient data (IPD) meta-analysis of randomised controlled trials (RCTs) and observational cohort studies to determine the clinical effectiveness of a delayed prescribing strategy on outcomes for respiratory tract infection, overall and for key subgroups of people.

## Methods

### Protocol and registration

The systematic review and IPD meta-analysis were performed according to the published study protocol that was registered with PROSPERO (CRD42018079400) and was reported in line with PRISMA-IPD (preferred reporting items for systematic reviews and meta-analysis of individual participant data).^19^ ^20^

### Eligibility criteria

We included all observational cohort studies and RCTs in a community setting that had a delayed antibiotic prescribing strategy (prescribed an antibiotic but advised the patient not to start taking the course unless their condition deteriorated or failed to improve after a set period), or a watchful waiting approach (observation for a set period to allow spontaneous symptom resolution before antibiotic prescription). Included studies also had a comparator group (no antibiotic prescription or immediate prescription). We excluded studies on antibiotic prescribing that were not RCTs or observational cohorts (eg, cross sectional, case-control, or survey studies), and studies on patients in hospital.

### Study identification and selection

Two researchers (HH and TB) searched the Cochrane Central Register of Controlled Trials, Ovid Medline, Ovid Embase, EBSCO CINAHL Plus, and Web of Science to identify eligible quantitative studies (observational cohort studies and RCTs). For observational studies, these searches were undertaken from inception to 23 October 2017. For RCTs, we updated the searches undertaken in the most recent Cochrane review, and searched from 26 May 2017 to 9 November 2017. We searched the International Standard Randomised Controlled Trial Number Registry, performed additional searches through Google, reviewed reference lists of identified papers, and contacted collaborators to identify any additional relevant studies. No language restrictions were reported. The full search strategy is available in the protocol.^19^ Searches were rerun on 8 October 2020 but no additional eligible studies were identified.

Two reviewers (HH and TB) independently screened titles and abstracts to determine inclusion criteria. Both reviewers independently assessed the full text of potentially relevant studies and determined eligibility. Discrepancies were resolved through discussion with a third reviewer (BS).

### Data collection processes

IPD were requested from the chief investigator for each eligible trial and observational study, initially by email and if no response was received after two emails by letter or telephone call. Once data had been received from the original authors, a complete database of all study data was prepared in Stata (version 15).^21^ TB and HH performed internal consistency checks against the published data to ensure the published analysis could be replicated. While the protocol contained a provision to contact study authors about any discrepancies, this did not prove necessary.

Data relating to the general characteristics of the study were extracted, such as study design, country, setting, type of respiratory tract infection, average age, and funding source. We requested all the variables that had been collected in the individual studies from the authors and received the full datasets. These variables were used in the observational studies to calculate the propensity score.

The IPD dataset included baseline data on prescribing strategy, age (0-4, 5-15, 16-65, and >65 years), fever at baseline consultation (greater than or less than 37.5ºC), previous duration of illness (above or below the median for each study), baseline severity of symptoms (average severity across all symptoms being above or below the median of each study), sex, smoking status (smoker or non-smoker), and lung disease (asthma, coronary obstructive pulmonary disease, or any other lung disease). Patients were classified as having a comorbidity if they had any of the chronic conditions (eg, heart disease, diabetes) for which data were collected in the original study. Follow-up data included symptom diaries (if collected) or days of illness determined by telephone interview, complications resulting in admission to hospital or death, reconsultation with same or worsening illness, and patient satisfaction.

### Risk of bias assessment for included studies

Two reviewers (HH and BS; TB and BS) independently assessed the risk of bias of each included study. RCTs were assessed using the Cochrane risk of bias tool for allocation bias (random sequence generation, allocation concealment, baseline imbalance), departures from intended interventions (participant and study personnel blinding, deviations from intended interventions, and analysis in groups to which they were randomised), attrition bias and appropriate methods to account for missing data, detection bias (blinding of outcome assessors), and selective outcome reporting.^22^ RCTs were considered to have a high risk of bias if scored as such in more than one of the six domains.

We assessed observational cohort studies using the ROBINS-I (risk of bias in non-randomised studies of interventions) tool for bias due to confounding, selection bias, bias due to deviations from intended intervention, and bias due to missing data and selective reporting.^23^ Observational cohort studies were considered to have a high risk of bias if judged to be at serious or critical risk of bias in at least one of the domains.

### Specification of outcome measures

The primary outcome of interest was the average symptom severity two to four days after the initial consultation. Symptom severity was measured on a seven item scale (0-6: normal, very little problem, slight problem, moderately bad, bad, very bad, as bad as could be).^24^ Secondary outcomes were duration of illness after the initial consultation, complications resulting in admission to hospital or death, reconsultation with the same or worsening illness, and patient satisfaction rated on a Likert scale. Reconsultation and complications (defined as hospital admission or death) were defined as binary outcomes (yes or no). Patient satisfaction data were rescaled to a four point scale to allow comparison across studies.^25^

### Synthesis methods

Study and patient level characteristics were described for all studies that contributed IPD. We performed a one stage IPD meta-analysis to obtain summary estimates and 95% confidence intervals for delayed antibiotic prescribing (compared with no antibiotic prescribing or immediate antibiotic prescribing) for each outcome measure.^18^ ^26^ The one stage approach combines all the data in a single meta-analysis based on a suitable regression model, with a random effect to account for individual studies. We used a linear regression to model the severity of symptoms and patient satisfaction, a count model to assess the duration of illness, and a logistic regression model to assess complications and reconsultation. All models controlled for baseline severity of illness, age, and condition (acute sore throat, cough or chest infection, otalgia or otitis media, or upper respiratory tract infection), and study type (RCT or observational). All participants were included as randomised and the primary analysis was of complete cases (without imputation for missing data).^27^

We used inverse probability weighting by propensity score analysis to adjust for baseline factor imbalance on measured covariates (such as age, sex, comorbid health conditions, and signs and symptoms at baseline consultation) in observational studies.^28^ ^29^ ^30^ Propensity scores based on covariates associated with any of the outcomes were derived for each observational study. We checked balance by using standardised mean differences and the appendix figures show the results. Propensity scores were also calculated for the RCTs by using the probability of randomised intervention given baseline covariates.^31^ An inverse probability of treatment weighting regression was carried out for the combined observational and RCT data to obtain a pooled estimate of treatment effect. We assessed heterogeneity across studies with the I^2^ statistic (tested by Higgins I^2^ test).^22^ Substantial statistical heterogeneity was considered to be present if the I^2^ statistic was greater than 50% and reasons for heterogeneity were explored.^22^

We repeated each model after including an interaction term between antibiotic prescribing strategy and subgroup characteristic to obtain summary estimates of the subgroup effects (interactions) of interest, which compared differential effects of interventions across the outcomes. The prespecified subgroups of interest were previous duration of illness (above or below median for the condition), age (<16, 16-64, >65 years), fever at baseline consultation (>37.5°C), comorbid conditions including lung comorbidity (such as asthma or chronic obstructive pulmonary disease), and severity of symptoms at baseline consultation.

We conducted several sensitivity analyses. All analyses were repeated using a two stage approach: IPD for each study were first analysed separately and then meta-analysed by using random effects models.^18^ We performed a two stage meta-analysis of extracted study level data from RCTs that did not contribute to the IPD to obtain summary estimates of effects of delayed antibiotic prescribing that combined IPD and non-IPD RCT studies, and to assess IPD availability bias.^32^ This process was not possible for observational studies because papers did not control consistently for the same confounding factors. Further sensitivity analyses included repeating the analyses after excluding studies with high risk of bias, and repeating subgroup analyses with age, fever, and baseline severity treated as continuous variables. All meta-analyses were undertaken with Stata software (version 15)^21^ and statistical significance was considered at the 5% level.

### Certainty of evidence per outcome

We used the five GRADE (grading of recommendations assessment, development and evaluation) considerations (study limitations, consistency of effect, imprecision, indirectness, and publication bias) to assess the quality of the evidence for our analysis of the primary outcomes.^33^

### Patient and public involvement

Two patient and public involvement team members (JB and KS) were involved in determining the research question, defining outcome measures, study design, and implementation. They attended all study meetings and are coauthors on this publication. We also shared our research findings with a patient and public involvement panel, allowing them to feedback to us their interpretation of the evidence and how general practitioners might more effectively communicate this information to patients. In the absence of published minimum clinically important differences for the outcomes considered in this study, it was particularly helpful to discuss their interpretation as to whether the differences observed represented a meaningful change. This feedback helped to inform our interpretation of the findings.

## Results

### Study selection and IPD obtained

**Fig 1:**
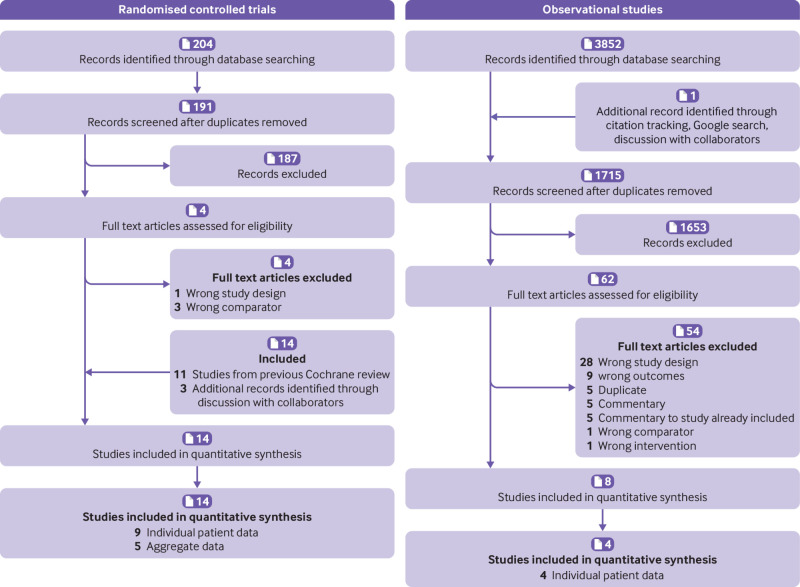
PRISMA (preferred reporting items for systematic reviews and meta-analysis) flowchart for randomised controlled trials and observational studies on delayed antibiotic prescribing

We sought IPD from 22 eligible studies (14 RCTs and eight observational studies), totalling 59 705 participants (fig 1).^6^ ^34^ ^35^ ^36^ ^37^ ^38^ ^39^ ^40^ ^41^ ^42^ ^43^ ^44^ ^45^ ^46^ ^47^ ^48^ ^49^ IPD were obtained from 13 studies (nine RCTs, four observational studies; table 1), totalling 55 682 participants. We were unable to obtain data from nine eligible studies because of no response (n=6), researchers moving on (n=2), or no response after initial agreement (n=1).^50^ ^51^ ^52^ ^53^ ^54^ ^55^ ^56^ ^57^ ^58^

**Table 1:** Characteristics of included studies

Reference	Country and setting	No and age of participants*	Condition	Intervention and comparison group	Type of delay	Outcome					Funding source
						SS	SD	R	C	PS	
**Randomised controlled trials**											
Little 1997	UK, primary care	716; 25.3 (17.0)	Sore throat	None, immediate, delayed	Prescription to be filled if symptoms did not start to settle after 3 days	+	+	+	+	−	Wessex NHS
Little 2001	UK, primary care	315; 5.0 (2.8)	Acute otitis media	None, immediate, delayed	Prescription collected after 72 hours if child still not improving	+	+	+	+	+	NHS R&D
Arroll 2002	New Zealand, primary care	129; 25.6 (23.0)	Common cold	Immediate, delayed	Prescription to be filled after 3 days if symptoms fail to improve	−	+	−	−	+	NZ Health Research Council
McCormick 2005	USA, paediatric clinic	223; 2.7 (2.7)	Acute otitis media	None, delayed	No antibiotics unless returning with acute ear symptoms within 30 days	+	+	+	+	+	US National Institutes of Health
Little 2005	UK, primary care	807; 39 (20.8)	Lower respiratory tract infection	None, immediate, delayed	Prescription to be filled if symptoms not resolved after 4 days	+	+	+	+	−	Medical Research Council
Chao 2008	USA, paediatric emergency department	232; 5.1 (2.4)	Acute otitis media	None, delayed	Advised to fill prescription if symptoms did not resolve in 2-3 days	−	+	+	−	+	NR
Little 2014	UK, primary care	889; 31.0 (21.2)	Acute respiratory tract infection	None, delayed	Four types: recontact, postdated, collection, patient led	+	+	+	+	+	National Institute for Health Research
De La Poza Abad 2016	Spain, primary care	405; 44.9 (16.6)	Acute respiratory tract infection	None, immediate, delayed	Collection or patient led if symptoms did not start to improve after a few days	+	+	+	+	+	Spanish Ministry of Health
Mas-Dalmau 2021	Spain, primary care	437; 6.3 (3.1)	Acute respiratory tract infection	None, immediate, delayed	Collection or patient led if symptoms did not start to improve after a few days	+	+	+	+	+	Spanish Ministry of Health
**Observational cohort studies**											
Butler, Francis et al 2012	13 European countries, primary care network	2690; 47.8 (16.3)	Cough or lower respiratory tract infection	None, immediate, delayed	Advised to fill if symptoms did not start to improve after 2-7 days	+	+	+	+	+	European Commission; National Institute for Health Research; Research Foundation-Flanders
Little 2013	UK, primary care	12 626; 34.0 (14.6)	Sore throat	None, immediate, delayed	Patient led	+	+	+	+	−	Medical Research Council; National Institute for Health Research
Little 2017	UK, primary care	28 856; 51.7 (17.9)	Acute lower respiratory tract infection	None, immediate, delayed	Advised to fill if symptoms did not start to improve; median advised delay=3 days	−	−	+	+	−	National Institute for Health Research
Hay 2016	UK, primary care	8320; 3.9 (3.7)	Acute cough and respiratory tract infection	None, immediate, delayed	Advised to fill if symptoms did not start to improve; median advised delay=3 days	+	+	+	+	−	National Institute for Health Research
**Studies for which individual patient data unavailable**											
Pichichero 1987	USA, paediatric clinic	114; 7.5 (2.6); 7.8 (2.3)	Sore throat	Immediate, delayed	No detail	+	+	+	−	−	RobertWood Johnson Foundation, Eli Lilly & Co, and Elmwood Paediatric Research fund
Gerber 1990	USA, paediatric clinic	113; NR	Sore throat	Immediate, delayed	No detail	+	−	−	−	−	NR
El-Daher 1991	Jordan, paediatric clinic	229; 7.8 (0.23); 8.3 (0.24)	Sore throat	Immediate, delayed	No detail	+	−	−	−	−	Biochemie GmbH and Jordan University of Science and Technology
Dowell 2001	UK, primary care	191; 41.6	Cough	Immediate, delayed	Patient to pick up prescription after 1 week of delay	−	+	+	−	+	Royal College of General Practitioners
Spiro 2006	USA, emergency department	283; 3.2	Acute otitis media	Immediate, delayed	No detail	−	+	−	−	−	US National Institutes of Health and Yale University School of Medicine
Siegel 2003	USA, paediatric clinic	194; 5	Acute otitis media	Immediate, delayed	To be filled only if symptoms did not improve within 2 days	−	−	−	−	−	Whitehall-Robins Healthcare
Marchetti 2005	Italy, primary care	1672; 4.8	Acute otitis media	Immediate, delayed	No detail	−	−	−	−	−	NR
Fischer 2009	USA, emergency department	144; NR	Acute otitis media	Immediate, delayed	To be filled only if symptoms did not improve within 2 days	−	−	−	−	−	NR
Kavanagh 2011	Ireland, primary care	120; 47.6 (16.3); 48 (17.8)	Acute cough or sore throat	Immediate, delayed	No detail	−	−	+	−	+	WestREN, MSD

C=complication; NR=nor reported; PS=patient satisfaction; R=reconsultation; SD=symptom duration; SS=symptom severity.

* Mean (standard deviation).

### Study characteristics

Each study included between 129 and 28 856 participants (median 557, interquartile range 316-2690). Participants belonged to a delayed antibiotic prescription group and an immediate or no antibiotic prescription group. Studies were conducted in the UK, the United States, New Zealand, Spain, and one study used data from multiple European studies. Most studies were conducted in primary care settings (n=11/13). Other settings included a paediatric emergency department (n=1) and paediatric clinic (n=1). Mean age of study participants ranged from 2.7 to 51.7 years. Six, four, and three studies examined all age groups, adult populations only, and paediatric populations only, respectively. One study focused on the common cold, two studies each assessed sore throat and cough, three focused on acute otitis media, and seven included more than one respiratory tract infection. Eleven out of 13 studies (84.6%) reported symptom severity and complication outcomes, 12 studies (92.3%) reported data on symptom duration and reconsultation, and eight studies (61.5%) reported patient satisfaction. Length of follow-up was 28-30 days.

Eligible studies that did not contribute IPD data (five trials and four observational studies) were generally smaller, based on younger populations, and had a higher proportion focused on acute otitis media and sore throat than IPD studies (table 2). Aggregate data were available for 930 patients from five RCTs that did not contribute IPD.

**Table 2:** Comparison of included and excluded study characteristics in individual patient data (IPD)

Eligible study characteristics	No (%) of studies	
	Included in IPD	Excluded from IPD
**Population source**		
Primary care	11 (84.6)	3 (33.3)
Emergency department	1 (7.7)	2 (22.2)
Paediatric office (USA)	1 (7.7)	4 (44.4)
**Condition**		
Common cold	1 (7.7)	0 (0.0)
Acute otitis media	3 (23.1)	4 (44.4)
Sore throat	2 (15.4)	4 (44.4)
Cough	2 (15.4)	2 (22.2)
Respiratory tract infection	7 (53.8)	0 (0.0)
**Antibiotic group**		
None	12 (92.3)	2 (22.2)
Immediate	11 (84.6)	7 (77.8)
Delayed	12 (92.3)	7 (77.8)

The mean age of IPD study participants was 38.7 years (table 3). Patients in the delayed antibiotics group were younger than those prescribed immediate antibiotics. A lower proportion of patients in the delayed antibiotic group had high baseline severity, longer previous duration of illness, fever at baseline, or lung disease compared with those in the immediate antibiotics group (table 3).

**Table 3:** Summary statistics for IPD database. Values are numbers (%) unless stated otherwise

Characteristics	Combined						RCTs						Observational studies				
	No of studies	No of participants	No antibiotics (n=19 360)	Immediate (n=28 129)	Delayed (n=8193)		No of studies	No of participants	No antibiotics (n=985)	Immediate (n=1082)	Delayed (n=1745)		No of studies	No of participants	No antibiotics (n=18 375)	Immediate (n=27 047)	Delayed (n=6448)
Age, years (mean (SD))	13	55 644	31.6 (23.4)	44.1 (22.3)	37.0 (22.4)		9	3779	27.2 (21.9)	23.4 (22.4)	24.2 (21.2)		4	51 865	31.8 (23.4)	44.9 (21.9)	40.4 (21.4)
Sex	12	54 848	—	—	—		8	2992	—	—	—		4	51 856	—	—	—
Male	—	—	7714 (40.4)	11 302 (40.6)	3047 (38.5)		—	—	281 (39.7)	352 (43.0)	621 (42.4)		—	—	7433 (40.5)	10 950 (40.5)	2426 (37.6)
Female	—	—	11 363 (59.6)	16 557 (59.4)	4865 (61.5)		—	—	427 (60.3)	466 (67.0)	845 (67.6)		—	—	10 936 (59.5)	16 091 (59.5)	4020 (62.4)
Condition	13	55 682	—	—	—		9	3812	—	—	—		4	51 870	—	—	—
Cough	4	—	14 067 (72.7)	21 497 (76.4)	5031 (61.4)		1	—	273 (27.7)	262 (24.2)	271 (15.5)		3	—	13 794 (75.1)	21 235 (78.5)	4760 (73.8)
Sore throat	2	—	4813 (24.9)	6058 (21.5)	1926 (23.5)		1	—	232 (23.6)	246 (22.7)	238 (13.6)		1	—	4581 (24.9)	5812 (21.5)	1688 (26.2)
RTI	4	—	364 (1.9)	311 (1.1)	846 (10.3)		4	—	364 (40.0)	311 (28.7)	846 (48.5)		—	—	—	—	—
Otitis media	3	—	116 (0.6)	263 (0.9)	390 (4.8)		3	—	116 (11.8)	263 (24.3)	390 (22.3)		—	—	—	—	—
Baseline severity average score, 0-4 (median (IQR))	13	54 956	0.7 (0.4-1.1)	0.9 (0.6-1.2)	0.8 (0.5-1.2)		8	3125	1.5 (0.75-2.0)	1.5 (0.75-2.1)	1.2 (0.8-1.8)		4	51 831	0.7 (0.4-1.0)	0.8 (0.6-1.2)	0.8 (0.5-1.1)
Below median	—	—	14 387 (75.2)	18 048 (64.7)	5616 (70.8)		—	—	400 (51.3)	490 (57.2)	895 (60.1)		—	—	13 987 (76.2)	17 558 (65.0)	4721 (73.2)
Median and above	—	—	4750 (24.8)	9837 (35.3)	2318 (29.2)		—	—	380 (48.7)	367 (42.8)	593 (39.9)		—	—	4370 (23.8)	9470 (35.0)	1725 (26.8)
Previous duration illness	11	54 462	—	—	—		6	2700	—	—	—		4	51 762	—	—	—
Below median	—	—	7794 (40.9)	10 388 (37.5)	3565 (46.3)		—	—	381 (52.0)	403 (57.4)	683 (53.9)		—	—	7413 (40.5)	9985 (37.0)	2882 (44.8)
Median and above	—	—	11 260 (59.1)	17 318 (62.5)	4137 (53.7)		—	—	351 (48.0)	299 (42.6)	583 (46.1)		—	—	10 909 (59.5)	17 019 (63.0)	3554 (55.2)
Fever at baseline	12	53 761	—	—	—		8	2896	—	—	—		4	50 865	—	—	—
No	—	—	11 169 (59.4)	13 472 (49.3)	4483 (58.8)		—	—	604 (79.7)	668 (81.7)	1002 (82.1)		—	—	10 565 (58.6)	12 804 (48.3)	3381 (53.7)
Yes	—	—	7634 (40.6)	13 865 (50.7)	3138 (41.2)		—	—	154 (20.3)	150 (18.3)	218 (17.9)		—	—	7480 (41.5)	13 715 (51.7)	2920 (46.3)
Any lung disease	11	47 504	—	—	—		7	2389	—	—	—		4	45 115	—	—	—
No	—	—	11 719 (80.1)	19 177 (74.2)	5618 (80.1)		—	—	514 (88.9)	560 (88.9)	1036 (87.7)		—	—	11 205 (79.7)	18 617 (73.8)	4582 (78.5)
Yes	—	—	2914 (19.9)	6679 (25.8)	1397 (19.9)		—	—	64 (11.1)	70 (11.1)	145 (12.3)		—	—	2850 (20.3)	6609 (26.2)	1252 (21.5)
Follow-up symptom severity score, 0-6 (median (IQR))	9	7074	1.8 (1-3)	2.03 (1.2-3.5)	2 (1.1-3.3)		6	2148	2.4 (1.4-3.8)	2.0 (0.8-3.0)	2.0 (1.0-3.0)		3	4926	1.7 (1.0-2.8)	2.1 (1.2-3.7)	2.0 (1.2-3.9)
Symptom duration, days (median (IQR))	11	8607	10 (5-28)	9 (4-28)	7 (3-12)		8	2918	5 (2-11)	6 (3-10)	6 (3-10)		3	5689	13 (6-28)	13 (5-28)	9 (4-28)
Reconsultation	11	54 105	—	—	—		8	2840	—	—	—		4	51 265	—	—	—
No	—	—	15 632 (83.2)	21 483 (77.3)	6433 (85.4)		—	—	556 (72.8)	678 (71.2)	842 (74.9)		—	—	15 076 (83.7)	20 805 (77.5)	5591 (87.2)
Yes	—	—	3155 (16.8)	6302 (22.7)	1100 (14.6)		—	—	208 (27.2)	274 (28.8)	282 (25.1)		—	—	2947 (16.4)	6028 (22.5)	818 (12.8)
Patient satisfaction score, 1-4 (median (IQR))	8	4584	4 (3-4)	4 (3-4)	3.2 (2.7-4)		—	2100	4 (2.7-4)	4 (3-4)	3.2 (2.7-4)		—	2484	3 (3-4)	4 (3-4)	4 (3-4)

IPD=individual patient data; IQR=interquartile range; RCT=randomised controlled trial; RTI=respiratory tract infection; SD=standard deviation.

### IPD integrity and risk of bias

**Fig 2:**
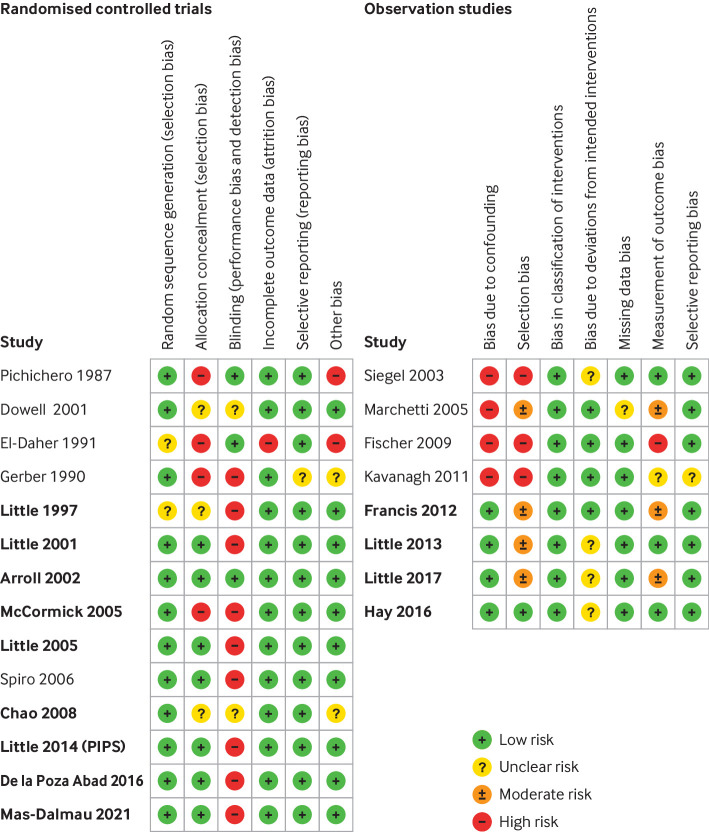
Risk of bias for randomised controlled trials (using the Cochrane risk of bias tool) and observation studies (risk of bias in non-randomised studies of interventions (ROBINS-I) tool). Bold studies contributed to individual patient data

For all included IPD, we were able to replicate aggregate results that were reported in each of the associated publications. Each individual study contributing IPD was deemed low or moderate risk of bias (fig 2), except for two RCTs that were judged to be high risk of bias on two domains. We also assessed the risk of bias of studies that did not contribute IPD. These studies were judged to have potentially high (n=6) or unclear (n=2) risk of bias (fig 2), and were more likely to be at risk of selection bias but also more likely to have been low risk of bias with respect to blinding.

### Mean symptom severity two to four days after consultation

**Fig 3:**
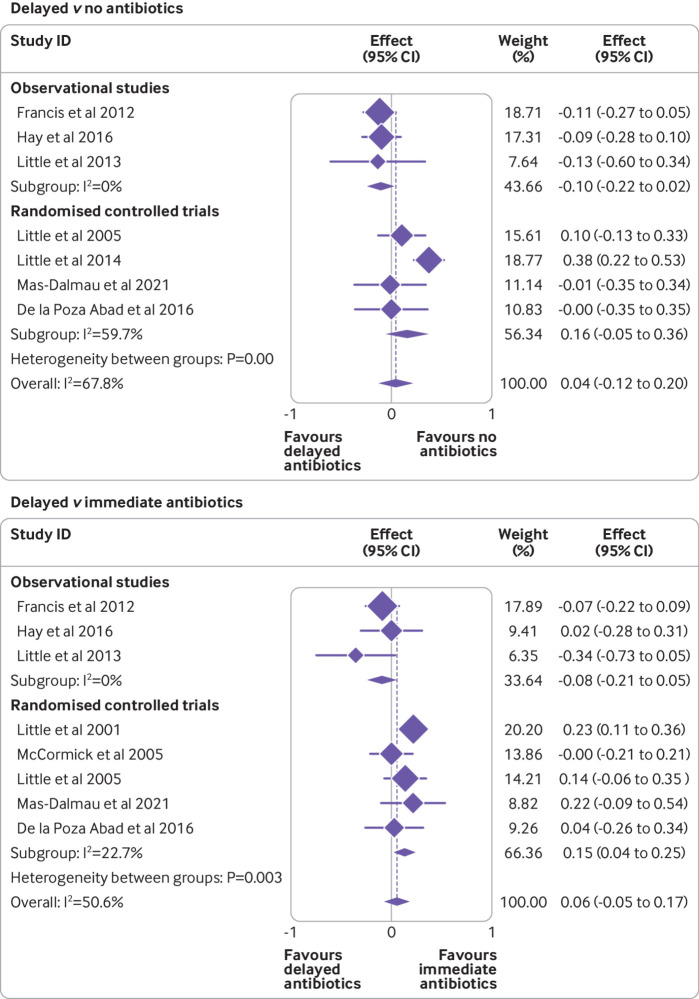
Unadjusted association between treatment group and symptom severity two to four days after consultation for delayed versus no antibiotics, and delayed versus immediate antibiotics. Weights are from random effects model

One stage random effects IPD meta-analysis of individual RCTs and observational studies combined found that, overall, there was no significant difference in symptom severity between delayed antibiotics and no antibiotics (mean difference on seven point scale −0.003, 95% confidence interval −0.12 to 0.11; seven studies, 3907 participants; table 4, fig 3). No significant difference was found in symptom severity between delayed and immediate antibiotics (0.02, −0.11 to 0.15; eight studies, 3752 participants; table 4, fig 3). Consistent results were obtained using a two stage approach.

**Table 4:** Effect of antibiotic prescribing strategy on all clinical outcomes in one stage random effects IPD meta-analysis. Values are means (95% confidence intervals) unless stated otherwise

Outcome	Delayed *v* no antibiotics						Delayed *v* immediate antibiotics				
	No of participants (none)	No of participants (delayed)	No antibiotics	Delayed	Adjusted* estimate (95% CI)		No of participants (immediate)	No of participants (delayed)	Immediate	Delayed	Adjusted* estimate (95% CI)
**Follow-up symptom severity score on days 2-4**											
RCT+OS	2716	1192	2.499 (1.44 to 3.56)	2.496 (1.47 to 3.52)	−0.003 (−0.12 to 0.11)		2699	1053	2.68 (1.26 to 4.11)	2.70 (1.36 to 4.05)	0.02 (−0.11 to 0.15)
RCT	484	815	2.77 (1.13 to 4.11)	2.86 (1.6 to 4.06)	0.09 (−0.10 to 0.28)		606	674	2.29 (0.66 to 3.93)	2.40 (0.78 to 4.03)	0.11 (−0.004 to 0.22)
OS	2232	377	2.22 (1.86 to 2.59)	2.12 (1.74 to 2.50)	−0.10 (−0.12 to −0.08)†		2093	379	2.75 (2.24 to 3.26)	2.63 (2.05 to 3.20)	−0.12 (−0.33 to 0.07)
**Time to complete symptom resolution (days)**											
RCT+OS	3091	1123	11.5 (6.4 to 16.6)	11.6 (6.4 to 16.6)	1.00 (0.82 to 1.23)‡		3275	1442	10.9 (6.3 to 15.6)	11.4 (6.8 to 15.9)	1.04 (1.01 to 1.08)†‡
RCT	540	647	9.42 (4.22 to 14.62)	9.88 (4.37 to 15.38)	1.04 (0.95 to 1.13)		876	962	9.94 (7.17 to 12.70)	11.37 (8.19 to 14.54)	1.14 (1.06 to 1.22)†
OS	2551	476	15.1 (14.8 to 15.4)	15.5 (15.2 to 15.8)	0.98 (0.94 to 1.01)		2399	480	11.96 (5.90 to 18.03)	12.37 (6.40 to 18.35)	1.02 (0.97 to 1.07)‡
**Duration of moderately severe or severe symptoms (days)**											
RCT+OS	2997	1251	6.2 (5.0 to 7.5)	6.3 (5.0 to 7.5)	1.01 (0.91 to 1.12)		2744	902	5.8 (4.5 to 7.2)	6.4 (5.3 to 7.6)	1.08 (1.01 to 1.17)†
RCT	447	775	6.88 (4.21 to 9.56)	6.81 (4.16 to 9.46)	1.04 (0.94 to 1.15)		347	426	6.36 (5.85 to 6.86)	7.48 (6.94 to 8.02)	1.18 (1.06 to 1.31)†‡
OS	2550	476	6.59 (5.45 to 6.74)	6.74 (6.60 to 6.87)	0.97 (0.93 to 1.01)		2397	476	5.36 (4.02 to 6.70)	5.94 (4.86 to 7.02)	1.05 (0.96 to 1.16)‡
**Reconsultation (%, 95% CI) **											
RCT+OS	16 232	5901	17 (4 to 58)	13 (2 to 52)	0.72 (0.60 to 0.87)†		22 430	6157	22 (4 to 58)	16 (3 to 59)	0.95 (0.74 to 1.22)
RCT	509	611	25 (15 to 22)	21 (10 to 31)	0.92 (0.67 to 1.26)		796	865	25 (5 to 41)	24 (8 to 41)	1.29 (0.84 to 1.99)
OS	15 723	5290	16 (4 to 60)	12 (2 to 54)	0.54 (0.49 to 0.60)†		21 634	5292	22 (4 to 58)	15 (3 to 59)	0.70 (0.66 to 0.75)†
**Complication (hospital admission or death; %, 95% CI)**											
RCT+OS	16 364	5827	0.6 (0.03 to 14)	0.4 (0.02 to 11)	0.62 (0.30 to 1.27)		22 371	6017	0.8 (0.1 to 5)	0.6 (0.1 to 4)	0.78 (0.53 to 1.13)
RCT	431	530	0.6 (0.02 to 14)	2.09 (0.01 to 22)	0.35 (0.07 to 1.92)		650	719	0.9 (0.3 to 5)	1.0 (0.3 to 4)	1.25 (0.38 to 4.16)
OS	15 933	5297	0.6 (0.01 to 3.7)	0.4 (0.01 to 2.2)	0.64 (0.28 to 1.43)		21 721	5298	0.7 (0.3 to 1.7)	0.6 (0.2 to 1.6)	0.22 (0.19 to 0.27)†
**Patient satisfaction score**											
RCT+OS	1434	674	2.96 (2.55 to 3.36)	3.04 (2.64 to 3.44)	0.09 (0.06 to 0.11)†		1743	805	2.99 (2.33 to 3.64)	2.87 (2.25 to 3.49)	−0.12 (−0.26 to 0.03)
RCT	433	520	3.17 (2.82 to 3.51)	3.26 (2.92 to 3.60)	0.06 (−0.03 to 0.16)		563	649	3.20 (2.33 to 4.06)	3.07 (2.22 to 3.92)	−0.13 (−0.31 to 0.05)
OS	1001	154	2.77 (2.15 to 3.39)	2.85 (2.25 to 3.46)	0.10 (−0.03 to 0.23)		1180	156	2.88 (2.07 to 3.69)	2.77 (2.01 to 3.52)	−0.06 (−0.18 to 0.06)

Adjusted estimate=coefficient, odds ratio, or relative risk; IPD=individual patient data; OS=observational study; RCT=randomised controlled trial.

* Adjusted for baseline severity, age, and condition.

† Statistically significant result. The number of observations for each outcome varies as not all studies collected data on all outcomes; analyses used all available data for each outcome.

‡ Estimated using fixed study specific effects and random treatment effect due to convergence difficulties.

### Subgroup effects

None of the prespecified subgroup variables modified the effectiveness of delayed antibiotic prescribing relative to no antibiotics (table 5). We found a significant overall interaction effect of age on the effectiveness of delayed relative to immediate antibiotic prescribing (mean difference −0.10, 95% confidence interval −0.17 to −0.03). Children younger than 5 years had a slightly higher follow-up symptom severity score two to four days after consultation with delayed versus immediate antibiotics (0.10, 0.03 to 0.18), whereas no significant difference was found in severity between delayed and immediate antibiotics for other age groups (table 5).

**Table 5:** Effect of antibiotic prescribing strategy subgroup variable interactions on mean symptom severity score two to four days after consultation for delayed versus no antibiotics, and delayed versus immediate antibiotics

Subgroup	No of studies	No of participants	Interaction (95% CI)	P	Adjusted* mean difference (95% CI)
**Delayed *v* no antibiotics**					
Previous duration					
Median and above	6	1835	0.05 (−0.23 to 0.34)	0.70	0.008 (−0.21 to 0.23)
Below median		1589			0.03 (−0.22 to 0.28)
Age (years)					
0-4	9	749	0.11 (−0.02 to 0.24)	0.11	−0.20 (−0.24 to −0.15)
5-15		637			0.12 (0.07 to 0.16)
16-64		2153			−0.03 (−0.14 to 0.08)
>65		368			0.07 (−0.36 to 0.51)
Fever					
≥37.5ºC	8	1436	0.01 (−0.16 to 0.18)	0.88	−0.03 (−0.15 to 0.09)
<37.5ºC		2211			−0.02 (−0.19 to 0.16)
Comorbidity					
Any lung disease	9	438	0.14 (−0.05 to 0.34)	0.15	0.15 (−0.12 to 0.42)
No lung disease		2598			−0.01 (−0.15 to 0.13)
Baseline severity					
Median and above	9	1972	−0.05 (−0.28 to 0.19)	0.69	−0.09 (−0.31 to 0.13)
Below median		1935			−0.14 (−0.33 to 0.04)
**Delayed *v* immediate antibiotics**					
Previous duration					
Median and above	6	1516	−0.07 (−0.41 to 0.27)	0.68	−0.04 (−0.38 to 0.29)
Below median		1526			0.02 (−0.17 to 0.21)
Age (years)					
0-4	9	729	−0.10 (−0.17 to −0.03)	0.005†	0.10 (0.03 to 0.18)
5-15		548			0.09 (−0.11 to 0.30)
16-64		2107			−0.09 (−0.27 to 0.09)
>65		366			−0.19 (−0.62 to 0.25)
Fever					
≥37.5ºC	8	1765	−0.05 (−0.32 to 0.23)	0.80	−0.01 (−0.42 to 0.40)
<37.5ºC		1662			0.03 (−0.06 to 0.11)
Comorbidity					
Any lung disease	9	483	0.01 (−0.16 to 0.19)	0.87	0.13 (−0.27 to 0.53)
No lung disease		2554			0.12 (−0.14 to 0.38)
Baseline severity					
Median and above	9	2286	0.35 (−0.23 to 0.93)	0.24	0.10 (−0.57 to 0.77)
Below median		1466			−0.27 (−0.34 to −0.19)

* Adjusted for baseline severity, age, and condition.

† Statistically significant interaction term.

### Secondary outcomes

Time to symptom resolution was longer with delayed (11.4 days) than immediate antibiotics (10.9 days; hazard ratio 1.04, 95% confidence interval 1.01 to 1.08). Reconsultation rates were lower with delayed (13%) than with no antibiotics (17%; odds ratio 0.72, 95% confidence interval 0.60 to 0.87), but were not statistically significantly different for delayed (16%) versus immediate antibiotics (22%; odds ratio 0.95, 95% confidence interval 0.74 to 1.22). Complications resulting in hospital admission or death were lower with delayed than with no antibiotics (odds ratio 0.62, 95% confidence interval 0.30 to 1.27) and lower in delayed than immediate antibiotics (0.78, 0.53 to 1.13), but neither result was statistically significant. Patient satisfaction was higher with delayed (3.04 points) than no antibiotics (2.96), but by a small difference (mean difference 0.09, 95% confidence interval 0.06 to 0.11; table 4).

### Quality of evidence across studies

Based on GRADE, the overall quality of the evidence for all outcomes in the IPD dataset was judged as moderate, apart from patient satisfaction which was low. Table 6 provides a full evidence profile. Two RCTs were deemed higher risk because of lack of blinding and allocation concealment, which lowered the rating for risk of bias to serious. However, consistent effects across RCTs suggest results are likely to be unbiased. Observational studies were considered high quality and achieved balance on potential covariates, but were downrated because residual confounding could not be ruled out.

**Table 6:** Evidence profiles based on GRADE (grading of recommendations assessment, development and evaluation) assessment

No of studies	Study design	Certainty assessment						No of participants		Adjusted* estimate (95% CI)	Quality	Importance
		Risk of bias	Inconsistency	Indirectness	Imprecision	Other considerations		No antibiotics	Delayed antibiotics			
**Delayed *v* no antibiotics**												
Symptom severity												
4	RCT	Serious†	Not serious‡	Not serious	Not serious§	None		484	815	0.09 (−0.10 to 0.28)	Moderate	Critical
3	OS	Serious¶	Not serious	Not serious	Not serious**	None		2231	377	−0.10 (−0.12 to −0.08)	Low	Critical
7	RCT+OS	Serious	Not serious	Not serious	Not serious§	None		2715	1192	−0.003 (−0.12 to 0.11)	Moderate	Critical
Time to complete symptom resolution (days)												
5	RCT	Serious†	Not serious‡	Not serious	Not serious§	None		540	647	1.04 (0.95 to 1.13)	Moderate	Important
3	OS	Serious¶	Not serious	Not serious	Not serious**	None		2551	476	0.98 (0.94 to 1.01)	Low	Important
8	RCT+OS	Serious	Not serious	Not serious	Not serious§	None		3091	1123	1.00 (0.82 to 1.23)	Moderate	Important
Reconsultation (%)												
5	RCT	Serious†	Not serious‡	Not serious	Not serious§	None		509	611	0.92 (0.67 to 1.26)	Moderate	Important
4	OS	Serious¶	Not serious	Not serious	Not serious**	None		15 723	5290	0.54 (0.49 to 0.60)	Low	Important
9	RCT+OS	Serious	Not serious	Not serious	Not serious§	None		16 232	5901	0.72 (0.60 to 0.87)	Moderate	Important
Complication (hospital admission or death; %)												
6	RCT	Serious†	Not serious‡	Not serious	Not serious§	None		431	530	0.35 (0.07 to 1.92)	Moderate	Important
4	OS	Serious¶	Not serious	Not serious	Not serious**	None		15 933	5297	0.60 (0.28 to 1.43)	Low	Important
10	RCT+OS	Serious	Not serious	Not serious	Not serious§	None		16 364	5827	0.62 (0.30 to 1.27)	Moderate	Important
Patient satisfaction score												
5	RCT	Serious†	Not serious‡	Not serious	Not serious§	None		433	520	0.06 (−0.03 to 0.16)	Moderate	Important
1	OS	Serious¶	NA	Not serious	Not serious**	None		1001	154	0.10 (−0.03 to 0.23)	Low	Important
6	RCT+OS	Serious	Not serious	Not serious	Not serious§	None		1434	674	0.09 (0.06 to 0.11)	Moderate	Important
**Delayed *v* immediate antibiotics**												
Symptom severity												
5	RCT	Serious†	Not serious	Not serious	Not serious	None		606	674	0.11 (−0.004 to 0.22)	Moderate	Critical
3	OS	Serious	Not serious	Not serious	Not serious	None		2093	377	−0.12 (−0.33 to 0.07)	Low	Critical
8	RCT+OS	Serious	Not serious	Not serious	Not serious	None		2699	1053	0.02 (−0.11 to 0.15)	Moderate	Critical
Time to complete symptom resolution (days)												
7	RCT	Serious	Serious‡	Not serious	Not serious	None		876	962	1.14 (1.06 to 1.22)	Low	Important
3	OS	Serious	Not serious	Not serious	Not serious	None		2399	480	1.02 (0.97 to 1.07)	Low	Important
10	RCT+OS	Serious	Not serious	Not serious	Not serious	None		3275	1442	1.04 (1.01 to 1.08)	Low	Important
Reconsultation (%)												
6	RCT	Serious	Not serious	Not serious	Not serious††	None		796	865	1.29 (0.84 to 1.99)	Moderate	Important
4	OS	Serious	Serious‡	Not serious	Not serious	None		21 634	5292	0.70 (0.66 to 0.75)	Low	Important
10	RCT+OS	Serious	Not serious	Not serious	Not serious	None		22 430	6157	0.95 (0.74 to 1.22)	Moderate	Important
Complication (hospital admission or death; %)												
3	RCT	Serious	Not serious	Not serious	Not serious††	None		650	719	1.25 (0.38 to 4.16)	Moderate	Important
4	OS	Serious	Not serious	Not serious	Not serious	None		21 721	5298	0.22 (0.19 to 0.27)	Low	Important
7	RCT+OS	Serious	Not serious	Not serious	Not serious	None		22 371	6017	0.78 (0.53 to 1.13)	Moderate	Important
Patient satisfaction score												
5	RCT	Serious	Serious‡	Not serious	Not serious	None		563	649	−0.13 (−0.31 to 0.05)	Low	Important
1	OS	Serious	NA	Not serious	Not serious	None		1180	156	−0.06 (−0.18 to 0.05)	Low	Important
6	RCT+OS	Serious	Serious	Not serious	Not serious	None		1743	805	−0.12 (−0.26 to 0.03)	Low	Important

Adjusted estimate=adjusted coefficient, odds ratio, or relative risk; NA=not applicable; OS=observational studies; RCT=randomised controlled trials.

*Adjusted for baseline severity, age, and condition.

†Most RCTs here were not blinded. However, results were not considered biased because similar evidence obtained for blinded studies and observational studies.

‡Statistical but not important heterogeneity.

§Confidence intervals exclude important benefits and harms.

¶Balance achieved for key covariates but residual confounding is still possible.

**Large enough sample size and the 95% confidence interval excludes no effect.

††Wide confidence intervals but not downgraded because overall same conclusion.

### Sensitivity analyses

*One stage versus two stage IPD analysis*—consistent results were observed for analyses using a two stage approach. We found no significant difference in symptom severity between delayed and no antibiotics (mean difference 0.04, 95% confidence interval −0.12 to 0.20) or between delayed and immediate antibiotics (0.06, −0.05 to 0.17).

*Exploring heterogeneity*—additional sensitivity analyses explored the effect of heterogeneity across studies. For symptom severity analyses, heterogeneity was found within the RCTs (I^2^=65%), and also between observational studies and RCTs (P<0.005, I^2^=68%), for delayed versus no antibiotics. The forest plots clearly showed that the results for Little (2014) were different from the other included studies, perhaps because it was the only study to test several delayed strategies in a single trial. When data from the Little study were excluded from the analyses, the heterogeneity within the RCTs was reduced (I^2^=0%), and also the overall heterogeneity between observational studies and RCTs was reduced (P=0.25, I^2^=0%). The results remained consistent with the main analysis (no significant difference in treatment effect). We did not observe any important variability for analyses that explored delayed versus immediate antibiotic comparison (heterogeneity between observational studies and RCTs; P=0.02, I^2^=24%).

*Subgroup analyses with continuous variables*—when we replaced dichotomised variables with a continuous variable for each subgroup, the subgroup results did not change. One exception was that patients with lower baseline severity had lower follow-up symptom severity with delayed versus immediate antibiotics (mean difference −0.27, 95% confidence interval −0.34 to −0.19).

**Fig 4:**
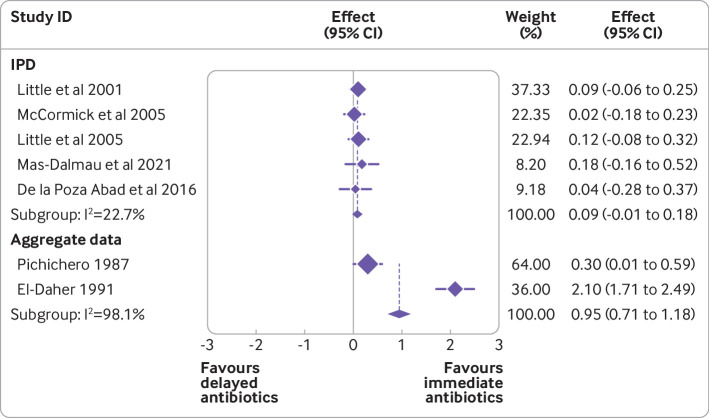
Difference in mean symptom severity two to four days after consultation; aggregate meta-analysis including studies that did not provide individual patient data (IPD)

*Including data from studies that did not provide IPD*—we carried out a further sensitivity analysis that included aggregate data from published estimates of studies that did not provide IPD. This sensitivity analysis compared the effect of immediate antibiotic prescribing with delayed antibiotic prescribing. We observed a stronger effect favouring immediate antibiotics for symptom severity (mean difference 0.95, 95% confidence interval 0.71 to 1.18) when including aggregate data, particularly the 1991 study by El-Daher, compared with IPD only analysis (0.09, −0.01 to 0.18; fig 4).

### Patient and public involvement

We approached our patient and public involvement panel of 10 people with a history of respiratory tract infections to discuss these results as they emerged. They agreed that the results were reassuring and did not suggest a meaningful benefit to taking antibiotics. They suggested that the way in which delayed prescribing is communicated to patients is important. They felt that some patients might not easily assess and gauge the severity or nature of their symptoms and would need clear guidance to determine whether they needed to take antibiotics. Almost all contributors emphasised that general practitioners need to be better at explaining the self-limiting nature of respiratory tract infections and the harmful effects of inappropriate use of antibiotics, using examples or pictures where necessary to relate to people and ensure a clear, simple, and effective message is delivered to patients. Qualitative studies of patients support these observations of our patient and public involvement collaborators.^59^

## Discussion

We used individual level data from 13 RCTs and observational cohort studies (55 682 patients) to assess the clinical effectiveness of delayed antibiotic prescribing in patients with respiratory tract infections in the community setting. Overall, our findings suggest delayed antibiotic prescribing is just as effective as no antibiotics for all clinical outcomes, but increased patient satisfaction and reduced reconsultation and complication rates. The reasons for reduced reconsultation rates are unclear, but one suggestion is that if a prescription is delayed, by the time the antibiotic course has finished, symptoms will have had more time to settle and so reconsultation is less likely; or it could be that secondary opportunistic bacterial infections that start later after an initial viral illness are more effectively managed by the later start of a delayed prescription. The second suggestion is supported by findings from the large GRACE trial; one of the groups that reported beneficial effects for antibiotics were people for whom evidence was found of coinfection with viruses and bacterial pathogens.^60^

Delayed antibiotics resulted in longer duration of symptoms than immediate antibiotics, but were as effective for the remaining clinical outcomes. The literature suggests that delayed prescribing could also reduce antibiotic use by patients compared with immediate antibiotics by 23-75%.^48^ ^61^ ^62^ Consistent results were obtained in subgroups often considered to be at higher risk, which suggests that delayed prescribing is unlikely to lead to poorer symptom control than immediate antibiotics. In children younger than 5 years and in those with higher symptom scores at baseline, we found statistically significant differences in the symptom severity scores two to four days after consultation. However, the mean differences were only 0.11 points higher on a scale from 0 to 6 (the equivalent of 1 in 10 participants rating symptoms one point different; for example, as moderately bad rather than a slight problem). This finding suggests that while the effect might be statistically significant, these differences are not clinically significant, and our patient and public involvement panel did not feel that they were likely to be meaningful to patients.

### Strengths and limitations

This large study examined the clinical effectiveness of the delayed antibiotic prescribing strategy. Strengths include the ability to control for baseline severity, to assess the quality of the studies based on the full dataset, to explore heterogeneity across studies, and to include results obtained from RCTs and observational studies. Selection bias associated with trials can limit perceived external validity, therefore a strength of this study was the ability to include observational data. Therefore, the external validity was improved and the impact of delayed antibiotic prescribing could be assessed in a clinical trial and a real world setting.^63^

The studies included in the IPD comprised 93% of the population from all eligible studies. The observational studies that did not provide data tended to be smaller studies. The trials for which IPD were not available but that were included in a sensitivity analysis were older studies (dating from 1991 or earlier). Therefore, the difference between the results of the primary analysis and the sensitivity analysis might be because the trials that did provide IPD are more likely to be relevant to modern patient populations. This difference might also be partly due to eligible trials that did not contribute IPD being based on younger populations, as highlighted in our subgroup analyses which showed that children younger than 5 years might benefit more from immediate antibiotics; however, this is unlikely because the size of the interaction was statistically significant but not clinically important. The studies that were included with aggregate data only were also at high risk of selection, attrition, and other biases. In particular, the study by El-Daher favoured immediate antibiotics over delayed antibiotics. The Cochrane review on this topic suggests that the El-Daher study was one of the less methodologically sound of the included studies. However, the El-Daher study is also the only one undertaken in a lower income setting^9^ and it is not clear whether the results of the IPD would generalise to that population. The illness spectrum in a lower income setting might be different, and the previous probability of more serious infection could be higher as could the risk of complications. Different organisms might be more prevalent and underlying comorbid conditions (such as tuberculosis) could lead to a different outcome. Delayed access to reassessment or secondary care in the event of deterioration might also be an important factor.^64^ Further research is needed in low to middle income countries to determine whether delayed antibiotic prescribing would be a safe and effective strategy in such settings.

A further limitation relates to the statistical power. Not all outcomes were collected in all studies. Symptom severity data were not collected for all studies, or were only collected for a subset of participants in some studies, resulting in a smaller sample size for the outcome analysis. This outcome was based on diary data and those who completed and returned diaries might not be representative of all study participants, which could also impact generalisability. However, previously published estimates from included studies suggest that those who completed diaries had broadly similar characteristics to all recruited participants.^65^ ^66^ Power was also low for the comparisons involving complications because this outcome is extremely rare, even in a dataset as large as the one we compiled. However, this extensive dataset enabled us to include large numbers of participants when analysing outcomes—even the smallest comparison contained 2108 participants—and the rarity of severe complications should be reassuring.

Delayed prescribing is one of several strategies that might help to safely reduce inappropriate antibiotic prescribing and consumption. Other strategies, such as point of care diagnostic testing, patient decision aids, and specific training for health professionals, might also be helpful alone or in combination with delayed prescribing.^67^ ^68^ However, none of the studies included in our IPD evaluated these strategies, which means we can only draw conclusions about delayed prescribing when used in isolation rather than in combination with other approaches that might be deployed in a primary care setting.

Looking across all the outcomes we included, we found a tendency for the treatment effect estimates from observational studies to be in the opposite direction from those of RCTs. This finding could be because of residual confounding (eg, use of other, or known or unmeasured covariates such as patient presence and compliance) in observational studies, differences in how delayed prescribing is implemented in real life versus RCTs, and varying time periods. However, the overall heterogeneity estimates for the combined RCT and observational study analyses were not high or could be explained by individual studies at higher risk of bias. We recognise that our pooled effect estimates were influenced by observational studies because these contributed large numbers of individual participants to the overall pooled dataset. The magnitude of the pooled treatment effect needs to be interpreted with caution because, while propensity scores were used to control for measured confounding, there might still be residual confounding from unmeasured confounders.

### Conclusions and implications

Delayed prescribing appears to be a safe and effective antibiotic strategy for most patients, including those in higher risk subgroups. Compared with a no prescription approach, delayed prescribing probably reduces reconsultation rates, and therefore the workload of general practitioners, with slightly higher levels of patient satisfaction. Compared with immediate antibiotics, delayed prescribing does not result in higher complication rates (if anything, they are lower) and it does not significantly decrease patient satisfaction. Delayed prescribing could be used as a standalone interventional approach, but it might also be a way of resolving mismatched expectations between clinician and patient.

**What is already known on this topic**

Clinical trials have suggested that delayed prescribing for respiratory tract infections is probably safe and effective for most patients
These clinical trials have been underpowered to look at subgroups or harms, and might be subject to selection bias

**What this study adds**

Individual patient data from randomised controlled trials and observational studies were used to investigate the effectiveness of delayed antibiotic prescribing (compared with no antibiotics or immediate antibiotic prescribing), overall and for subgroups such as children and those with comorbidities
Delayed prescribing was associated with similar symptom severity and duration as no antibiotics, but patient satisfaction was higher and reconsultation rates were lower; the effectiveness did not differ for any of the high risk subgroups
Delayed prescribing is unlikely to lead to poorer symptom control than immediate prescribing; older age was associated with increasing benefit on symptom severity two to four days after consultation

## Web extra

Extra material supplied by authors

## Notes

Contributors: BS and HH contributed equally to this paper. BS is the guarantor. BS conceived the original study concept and design. BS, PL, MM, SZ, GY, DB, JB, KS, and HCB wrote the grant application and obtained funding from the National Institute for Health Research (NIHR) Research for Patient Benefit (RfPB) Programme. TB, HH, BS, DB, and HCB contributed to the data analysis. BS, HH, and TB wrote the first draft of the manuscript. All listed authors contributed to the concept and design of the study, the interpretation of the results, and manuscript editing. All authors read, provided feedback, and approved the final manuscript. The corresponding author attests that all listed authors meet authorship criteria and that no others meeting the criteria have been omitted.

Ethical approval: Ethical approval was obtained by the Faculty of Medicine Ethics Committee at the University of Southampton (approval No 30068).

Data sharing: The IPD database can be shared subject to the approval of the study collaborators.

The lead author affirms that the manuscript is an honest, accurate, and transparent account of the study being reported; that no important aspects of the study have been omitted; and that any discrepancies from the study as planned have been explained.

Dissemination to participants and related patient and public communities: The findings of the study were disseminated to all members of the patient and public involvement panel at the time of our discussion with them as reported in this paper. No members asked for further disseminated information.

Provenance and peer review: Not commissioned; externally peer reviewed.

## References

[1] Ridge KW, Hand K, Sharland M, et al. Antimicrobial resistance. In: Chief Medical Officer annual report 2011: antimicrobial resistance. 2011. http://www.dh.gov.uk/health/2013/03/cmo-vol2/.

[2] Public Health England. English surveillance programme for antimicrobial utilisation and resistance report. https://assets.publishing.service.gov.uk/government/uploads/system/uploads/attachment_data/file/843129/English_Surveillance_Programme_for_Antimicrobial_Utilisation_and_Resistance_2019.pdf (accessed 7 Jul 2020).

[3] Cassini A Högberg LD Plachouras D Burden of AMR Collaborative Group. Attributable deaths and disability-adjusted life-years caused by infections with antibiotic-resistant bacteria in the EU and the European Economic Area in 2015: a population-level modelling analysis. Lancet Infect Dis 2019;19:56-66. 10.1016/S1473-3099(18)30605-4. 30409683 PMC6300481

[4] Organisation for Economic Cooperation and Development. Stemming the Superbug Tide. OECD, 2018.

[5] Costelloe C Metcalfe C Lovering A Mant D Hay AD. Effect of antibiotic prescribing in primary care on antimicrobial resistance in individual patients: systematic review and meta-analysis. BMJ 2010;340:c2096. 10.1136/bmj.c2096. 20483949

[6] Little P Stuart B Hobbs FDR DESCARTE investigators. Antibiotic prescription strategies for acute sore throat: a prospective observational cohort study. Lancet Infect Dis 2014;14:213-9. 10.1016/S1473-3099(13)70294-9. 24440616

[7] Spinks A Glasziou PP Del Mar CB. Antibiotics for sore throat. Cochrane Database Syst Rev 2013;2013:CD000023. 10.1002/14651858.CD000023.pub4. 24190439 PMC6457983

[8] Kenealy T Arroll B. Antibiotics for the common cold and acute purulent rhinitis. Cochrane Database Syst Rev 2013;2013:CD000247. 10.1002/14651858.CD000247.pub3. 16034850

[9] Spurling GKP Del Mar CB Dooley L Foxlee R Farley R. Delayed antibiotic prescriptions for respiratory infections. Cochrane Database Syst Rev 2017;9:CD004417. 10.1002/14651858.CD004417.pub5. 28881007 PMC6372405

[10] The path of least resistance: main report/Standing Medical Advisory Committee Sub-Group on Antimicrobial Resistance. Department of Health. 1998.

[11] National Institute for Health and Care Excellence. Respiratory tract infections (self-limiting): prescribing antibiotics Clinical guideline. 2008. www.nice.org.uk/guidance/cg69 (accessed 7 Jul 2020). 31815394

[12] Gulliford MC Dregan A Moore MV. Continued high rates of antibiotic prescribing to adults with respiratory tract infection: survey of 568 UK general practices. BMJ Open 2014;4:e006245. 10.1136/bmjopen-2014-006245. 25348424 PMC4212213

[13] Pouwels KB Dolk FCK Smith DRM Robotham JV Smieszek T. Actual versus ‘ideal’ antibiotic prescribing for common conditions in English primary care. J Antimicrob Chemother 2018;73(suppl_2):19-26. 10.1093/jac/dkx502. 29490060 PMC5890776

[14] Smieszek T Pouwels KB Dolk FCK. Potential for reducing inappropriate antibiotic prescribing in English primary care. J Antimicrob Chemother 2018;73(suppl_2):ii36-43. 10.1093/jac/dkx500. 29490058 PMC5890667

[15] Fiore DC Fettic LP Wright SD Ferrara BR. Antibiotic overprescribing: still a major concern. J Fam Pract 2017;66:730-6. 29202142

[16] van der Velden A Duerden M Bell J. Prescriber and patient responsibilities in treatment of acute respiratory tract infections—essential for conservation of antibiotics. Antibiotics (Basel) 2013;2:316-27. 10.3390/antibiotics2020316.

[17] Lambert PC Sutton AJ Abrams KR Jones DR. A comparison of summary patient-level covariates in meta-regression with individual patient data meta-analysis. J Clin Epidemiol 2002;55:86-94. 10.1016/S0895-4356(01)00414-0. 11781126

[18] Riley RD Lambert PC Abo-Zaid G. Meta-analysis of individual participant data: rationale, conduct, and reporting. BMJ 2010;340:c221. 10.1136/bmj.c221. 20139215

[19] Stuart B Hounkpatin H Becque T. Delayed antibiotic prescribing for respiratory tract infections: protocol of an individual patient data meta-analysis. BMJ Open 2019;9:e026925. 10.1136/bmjopen-2018-026925. 30670532 PMC6347865

[20] Stewart LA Clarke M Rovers M. Preferred Reporting Items for a Systematic Review and Meta-analysis of Individual Participant Data: The PRISMA-IPD Statement. JAMA 2015;313;1657-65. 10.1001/jama.2015.3656. 25919529

[21] StataCorp. Stata Statistical Software: Release 15. 2017.

[22] Higgins JPT Altman DG Gøtzsche PC Cochrane Bias Methods Group Cochrane Statistical Methods Group. The Cochrane Collaboration’s tool for assessing risk of bias in randomised trials. BMJ 2011;343:d5928. 10.1136/bmj.d5928. 22008217 PMC3196245

[23] Sterne JA Hernán MA Reeves BC. ROBINS-I: a tool for assessing risk of bias in non-randomised studies of interventions. BMJ 2016;355:i4919. 10.1136/bmj.i4919. 27733354 PMC5062054

[24] Watson L Little P Moore M Warner G Williamson I. Validation study of a diary for use in acute lower respiratory tract infection. Fam Pract 2001;18:553-4. 10.1093/fampra/18.5.553. 11604383

[25] Schünemann HJ Vist GE Higgins JP. Interpreting results and drawing conclusions. In: Cochrane Handbook for Systematic Reviews of Interventions. Wiley, 2019: 403-31, 10.1002/9781119536604.ch15.

[26] Stewart GB Altman DG Askie LM Duley L Simmonds MC Stewart LA. Statistical analysis of individual participant data meta-analyses: a comparison of methods and recommendations for practice. PLoS One 2012;7:e46042. 10.1371/journal.pone.0046042. 23056232 PMC3463584

[27] Akl EA Kahale LA Agoritsas T. Handling trial participants with missing outcome data when conducting a meta-analysis: a systematic survey of proposed approaches. Syst Rev 2015;4:98. 10.1186/s13643-015-0083-6. 26202162 PMC4511978

[28] US Government Accountability Office. Cross-design synthesis: a new strategy for medical effectiveness research. 1992. https://www.gao.gov/products/PEMD-92-18 (accessed 7 Jul 2020).

[29] Rosenbaum PR Rubin DB. The central role of the propensity score in observational studies for causal effects. Biometrika 1983;60:41-55. 9802183

[30] D’Agostino RB Jr. Propensity score methods for bias reduction in the comparison of a treatment to a non-randomized control group. Stat Med 1998;17:2265-81. 10.1002/(SICI)1097-0258(19981015)17:19<2265::AID-SIM918>3.0.CO;2-B. 9802183

[31] Williamson EJ Forbes A White IR. Variance reduction in randomised trials by inverse probability weighting using the propensity score. Stat Med 2014;33:721-37. 10.1002/sim.5991. 24114884 PMC4285308

[32] Riley RD Simmonds MC Look MP. Evidence synthesis combining individual patient data and aggregate data: a systematic review identified current practice and possible methods. J Clin Epidemiol 2007;60:431-9. 10.1016/j.jclinepi.2006.09.009. 17419953

[33] Mustafa RA Santesso N Brozek J. The GRADE approach is reproducible in assessing the quality of evidence of quantitative evidence syntheses. J Clin Epidemiol 2013;66:736-42, quiz 742.e1-5. 10.1016/j.jclinepi.2013.02.004. 23623694

[34] Little P Williamson I Warner G. Open randomised trial of prescribing strategies in managing sore throat. *BMJ* 1997;314:722-7. 10.1136/bmj.314.7082.722 PMC2126131 9116551

[35] Francis NA Gillespie D Nuttall J. Delayed antibiotic prescribing and associated antibiotic consumption in adults with acute cough. Br J Gen Pract 2012;62:e639-46. 10.3399/bjgp12X654614. 22947585 PMC3426603

[36] Little P Stuart B Hobbs FD DESCARTE investigators. Predictors of suppurative complications for acute sore throat in primary care: prospective clinical cohort study. BMJ 2013;347:f6867. 10.1136/bmj.f6867. 24277339 PMC3898431

[37] Moore M Stuart B Hobbs FR DESCARTE investigators. Symptom response to antibiotic prescribing strategies in acute sore throat in adults: the DESCARTE prospective cohort study in UK general practice. Br J Gen Pract 2017;67:e634-42. 10.3399/bjgp17X692321. 28808075 PMC5569743

[38] Hay AD Redmond NM Turnbull S. Development and internal validation of a clinical rule to improve antibiotic use in children presenting to primary care with acute respiratory tract infection and cough: a prognostic cohort study. Lancet Respir Med 2016;4:902-10. 10.1016/S2213-2600(16)30223-5. 27594440 PMC5080970

[39] Little P Stuart B Smith S. Antibiotic prescription strategies and adverse outcome for uncomplicated lower respiratory tract infections: prospective cough complication cohort (3C) study. BMJ 2017;357:j2148. 10.1136/bmj.j2148. 28533265 PMC5439222

[40] Mas-Dalmau G Villanueva López C Gorrotxategi Gorrotxategi P DAP PEDIATRICS GROUP*. Delayed antibiotic prescription for children with respiratory infections: A Randomized Trial. Pediatrics 2021;147:e20201323. 33574163 10.1542/peds.2020-1323

[41] Butler CC Hood K Verheij T. Variation in antibiotic prescribing and its impact on recovery in patients with acute cough in primary care: prospective study in 13 countries. BMJ 2009;338:b2242. 10.1136/bmj.b2242. 19549995 PMC3272656

[42] Little P Gould C Williamson I Warner G Gantley M Kinmonth AL. Reattendance and complications in a randomised trial of prescribing strategies for sore throat: the medicalising effect of prescribing antibiotics. BMJ 1997;315:350-2. 10.1136/bmj.315.7104.350. 9270458 PMC2127265

[43] Little P Gould C Williamson I Moore M Warner G Dunleavey J. Pragmatic randomised controlled trial of two prescribing strategies for childhood acute otitis media. BMJ 2001;322:336-42. 11159657 10.1136/bmj.322.7282.336 PMC26576

[44] Arroll B Kenealy T Kerse N. Do delayed prescriptions reduce the use of antibiotics for the common cold? A single-blind controlled trial. J Fam Pract 2002;51:324-8. 11978254

[45] McCormick DP Chonmaitree T Pittman C. Nonsevere acute otitis media: a clinical trial comparing outcomes of watchful waiting versus immediate antibiotic treatment. Pediatrics 2005;115:1455-65. 10.1542/peds.2004-1665. 15930204

[46] Little P Rumsby K Kelly J. Information leaflet and antibiotic prescribing strategies for acute lower respiratory tract infection: a randomized controlled trial. JAMA 2005;293:3029-35. 10.1001/jama.293.24.3029. 15972565

[47] Chao JH Kunkov S Reyes LB Lichten S Crain EF. Comparison of two approaches to observation therapy for acute otitis media in the emergency department. Pediatrics 2008;121:e1352-6. 10.1542/peds.2007-2278. 18450878

[48] Little P Moore M Kelly J PIPS Investigators. Delayed antibiotic prescribing strategies for respiratory tract infections in primary care: pragmatic, factorial, randomised controlled trial. BMJ 2014;348:g1606. 10.1136/bmj.g1606. 24603565 PMC3944682

[49] de la Poza Abad M Mas Dalmau G Moreno Bakedano M Delayed Antibiotic Prescription (DAP) Group. Prescription strategies in acute uncomplicated respiratory infections: a randomized clinical trial. JAMA Intern Med 2016;176:21-9. 10.1001/jamainternmed.2015.7088. 26719947

[50] Pichichero ME Disney FA Talpey WB. Adverse and beneficial effects of immediate treatment of Group A beta-hemolytic streptococcal pharyngitis with penicillin. Pediatr Infect Dis J 1987;6:635-43. 10.1097/00006454-198707000-00004 3302916

[51] Gerber MA Randolph MF DeMeo KK Kaplan EL. Lack of impact of early antibiotic therapy for streptococcal pharyngitis on recurrence rates. J Pediatr 1990;117:853-8. 2123239 10.1016/s0022-3476(05)80121-0

[52] el-Daher NT Hijazi SS Rawashdeh NM al-Khalil IA Abu-Ektaish FM Abdel-Latif DI. Immediate vs. delayed treatment of group A beta-hemolytic streptococcal pharyngitis with penicillin V. Pediatr Infect Dis J 1991;10:126-30. 10.1097/00006454-199102000-00010 1905799

[53] Dowell J Pitkethly M Bain J Martin S. A randomised controlled trial of delayed antibiotic prescribing as a strategy for managing uncomplicated respiratory tract infection in primary care. Br J Gen Pract 2001;51:200-5. 11255901 PMC1313951

[54] Spiro DM Tay KY Arnold DH Dziura JD Baker MD Shapiro ED. Wait-and-see prescription for the treatment of acute otitis media: a randomized controlled trial. JAMA 2006;296:1235-41. 10.1001/jama.296.10.1235. 16968847

[55] Siegel RM Kiely M Bien JP. Treatment of otitis media with observation and a safety-net antibiotic prescription. Pediatrics 2003;112:527-31. 10.1542/peds.112.3.527 12949278

[56] Marchetti F Ronfani L Nibali SC Tamburlini G Italian Study Group on Acute Otitis Media. Delayed prescription may reduce the use of antibiotics for acute otitis media: a prospective observational study in primary care. Arch Pediatr Adolesc Med 2005;159:679-84. 10.1001/archpedi.159.7.679. 15997003

[57] Fischer T Singer AJ Chale S. Observation option for acute otitis media in the emergency department. Pediatr Emerg Care 2009;25:575-8. https://journals.lww.com/pec-online/Fulltext/2009/09000/Observation_Option_for_Acute_Otitis_Media_in_the.8.aspx. 10.1097/PEC.0b013e3181b91ff0 19755891

[58] Kavanagh KE O’Shea E Halloran R Cantillon P Murphy AW. A pilot study of the use of near-patient C-reactive protein testing in the treatment of adult respiratory tract infections in one Irish general practice. BMC Fam Pract 2011;12:93. 10.1186/1471-2296-12-93. 21880122 PMC3175160

[59] McDermott L Leydon GM Halls A PIPS investigators. Qualitative interview study of antibiotics and self-management strategies for respiratory infections in primary care. BMJ Open 2017;7:e016903. 10.1136/bmjopen-2017-016903. 29180593 PMC5719297

[60] Bruyndonckx R Stuart B Little P GRACE project group. Amoxicillin for acute lower respiratory tract infection in primary care: subgroup analysis by bacterial and viral aetiology. Clin Microbiol Infect 2018;24:871-6. 10.1016/j.cmi.2017.10.032. 29108950 PMC7128813

[61] Hungenberg M Hansen-Guzman A Jankousky KC. Does a delayed antibiotic prescribing strategy for respiratory infections result in fewer prescriptions compared with an immediate prescribing or no-prescribing strategy? Evidence-Based Pract. 2019;22:1-2. 10.1097/EBP.0000000000000508.

[62] Høye S Frich JC Lindbæk M. Use and feasibility of delayed prescribing for respiratory tract infections: a questionnaire survey. BMC Fam Pract 2011;12:34. 10.1186/1471-2296-12-34. 21592334 PMC3114766

[63] Faraoni D Schaefer ST. Randomized controlled trials vs. observational studies: why not just live together? BMC Anesthesiol 2016;16:102. 10.1186/s12871-016-0265-3. 27769172 PMC5073487

[64] Jehan F Nisar I Kerai S. Randomized trial of amoxicillin for pneumonia in Pakistan. N Engl J Med 2020;383:24-34. 10.1056/NEJMoa1911998. 32609980 PMC7244232

[65] Moore M Stuart B Hobbs FR DESCARTE investigators. Symptom response to antibiotic prescribing strategies in acute sore throat in adults: the DESCARTE prospective cohort study in UK general practice. Br J Gen Pract 2017;67:e634-42. 10.3399/bjgp17X692321. 28808075 PMC5569743

[66] Wensaas KA Heron J Redmond N. Post-consultation illness trajectories in children with acute cough and respiratory tract infection: prospective cohort study. Fam Pract 2018;35:676-83. 10.1093/fampra/cmy021. 29897430 PMC6290772

[67] Moore M. Antibiotics: time to act. Br J Gen Pract 2013;63:340-1. 10.3399/bjgp13X668447. 23834861 PMC3693776

[68] Cooke J Butler C Hopstaken R. Narrative review of primary care point-of-care testing (POCT) and antibacterial use in respiratory tract infection (RTI). BMJ Open Respir Res 2015;2:e000086. 10.1136/bmjresp-2015-000086. 25973210 PMC4426285

