# Supplementary material for: Delayed antibiotic prescribing for respiratory tract infections: individual patient data meta-analysis
Source: BMJ. 2021 Apr 28;373:n808. doi: 10.1136/bmj.n808 (PMC8080136; doi:10.1136/bmj.n808)
Supplement: Supplementary file 1 — Supplementary information: appendix figures [file stub062923.ww.docx]

Appendix Figures – Covariate imbalance prior and after adjustment using propensity scores for each observational study

Butler et al., Francis et al 2012 – (a) delayed vs none and (b) delayed vs immediate

(a) (b)

Hay et al 2016 (a) delayed vs none and (b) delayed vs immediate

(a)

(b)

Little et al 2013 (a) delayed vs none and (b) delayed vs immediate

1. (b)

Little et al 2017 (a) delayed vs none and (b) delayed vs immediate

1.

(b)
